# A novel difficulty grading system for laparoscopic living donor nephrectomy

**DOI:** 10.1007/s00464-020-07727-w

**Published:** 2020-06-15

**Authors:** Kosei Takagi, Hendrikus J. A. N. Kimenai, Turkan Terkivatan, Khe T. C. Tran, Jan N. M. Ijzermans, Robert C. Minnee

**Affiliations:** 1 Department of Surgery, Division of HPB & Transplant Surgery, University Medical Centre Rotterdam, Dr. Molewaterplein 40, Erasmus MC 3015 GD Rotterdam, The Netherlands; 2 Department of Gastroenterological Surgery, Dentistry, and Pharmaceutical Sciences, Okayama University Graduate School of Medicine, Okayama, Japan

**Keywords:** Kidney transplantation, Living donors, Nephrectomy, Laparoscopy, Hand-assisted laparoscopy, Learning curve, Education, Teaching

## Abstract

**Background:**

Several difficulty grading systems have been developed as a useful tool for selecting patients and training surgeons in laparoscopic procedures. However, there is little information on predicting the difficulty of laparoscopic donor nephrectomy (LDN). The aim of this study was to develop a grading system to predict the difficulty of LDN.

**Methods:**

Data of 1741 living donors, who underwent pure or hand-assisted LDN between 1994 and 2018 were analyzed. Multivariable analyses were performed to identify factors associated with prolonged operative time, defined as a difficulty index with 0 to 8. The difficulty of LDN was classified into three levels based on the difficulty index.

**Results:**

Multivariable analyses identified that male (odds ratio [OR] 1.69, 95% CI 1.37–2.09, *P* < 0.001), BMI > 28 (OR 1.36, 95% CI 1.08–1.72, *P* = 0.009), pure LDN (OR 1.99, 95% CI 1.53–2.60, P < 0.001), multiple renal arteries (OR 2.38, 95% CI 1.83–3.10, *P* < 0.001) and multiple renal veins (OR 2.18, 95% CI 1.52–3.16, *P* < 0.001) were independent risk factors influencing prolonged operative time. The difficulty index based on these factors was calculated and categorized into three levels: low (0–2), intermediate (3–5), and high (6–8) difficulty. Operative time was significantly longer in the high difficulty group (225 min) than in the low (169 min, *P* < 0.001) and intermediate difficulty group (194 min, *P* < 0.001). The conversion rate was higher in the high difficulty group (4.4%) than in the low (2.1%, *P* = 0.04) and the intermediate difficulty group (3.0%, *P* = 0.27). No significant difference in major complications was found between the groups.

**Conclusion:**

We developed a novel grading system with simple preoperative donor factors to predict the difficulty of LDN. This grading system may help surgeons in patient selection to advance their experiences and/or teach fellows from simple to difficult LDN.

The advantages of minimally invasive surgery over open donor nephrectomy (DN) are well established; reductions in length of stay, pain, and convalescence; faster return to normal activity [1]. Pure and hand-assisted laparoscopic donor nephrectomy (LDN) are common modalities of minimally invasive surgery [2]. Although these procedures have been introduced as standard surgical techniques in transplant centers throughout the world, it is important for surgeons to gradually increase surgical skills in accordance with their experience level and in combination with surgical difficulty influenced by donor factors [3, 4].

Recently, several practical difficulty scoring systems have been developed in the field of laparoscopic procedures [5–7], and can be helpful for selecting patients and educating surgeons especially for those with initial laparoscopic experiences. In addition, these scoring systems can quantify the degree of difficulty creating more awareness for more complex cases and their pitfalls in experienced surgeons. LDN can be harmful for healthy individuals, therefore accurate assessment of the degree of difficulty is crucial. Previous studies with small patient cohorts have reported the effectiveness of assessing preoperative imaging to predict technical difficulty in LDN [8, 9], however their models were complex requiring radiologists and special software. Therefore, a simpler difficulty grading system based on preoperative donor characteristics should be constructed using a large patient cohort.

The aim of the present study was to develop a grading system to predict the difficulty of LDN in a high-volume center of living kidney donation and kidney transplantation in Western Europe. Moreover, we aimed to propose the benchmark operation of LDN.

## Material and methods

### Patients

A retrospective review was performed using a prospective kidney transplant database including 1741 consecutive living donors who underwent LDN at the Erasmus MC, University Medical Centre Rotterdam, The Netherlands, between January 1998 and December 2018. The present study was approved by the Ethics Committee of the Erasmus MC (MEC-2019-0373), and was conducted in accordance with the tenets of the Declaration of Helsinki. This type of retrospective analysis does not require informed consent from the individual patients.

Using the kidney transplant database, the following donor data were collected: age, gender, body mass index (BMI), relationship between donors and recipients, the technique of LDN (pure or hand-assisted), the side of LDN (right or left kidney), operative time, blood loss, conversion rate, number of renal artery (single or multiple), number of renal vein (single or multiple), the incidence of postoperative major complications (Clavien-Dindo grade ≥ III [10]), and postoperative length of stay (LOS). A conversion from LDN to hand-assisted DN was considered as a conversion in this study. Postoperative major complications were defined as complications requiring radiological or surgical intervention, and life threatening complications [10].

### Surgical technique

Details of surgical techniques of LDN were previously reported [11]. Donors were placed in right- or left- decubitus positon. Pure LDN was performed with 4 or 5 trocars with carbon dioxide to 12-cm H^2^O pressure and a 30° video endoscope. The colon was mobilized, and perirenal fat was divided using an ultrasonic device (Harmonic, Ethicon, Cincinnati, USA). The ureter, the renal artery, and the renal vein were identified and dissected, afterwards a Pfannenstiel incision was made. The ureter, the renal artery, and renal vein were divided and the donor kidney was extracted using the endobag through the Pfannenstiel incision. In case of conversion from LDN to hand-assisted DN, a Gelport (Applied Medical, Rancho Santa Margarita, California, USA) was inserted via the Pfannenstiel incision.

Hand-assisted LDN were performed through the retroperitoneoscopic approach. First a Pfannenstiel incision was made to create a retroperitoneal space and a Gelport was inserted. Three trocars were replaced with a triangular shape, and carbon dioxide was insufflated retroperitoneally to 12-cm H^2^O pressure. Dissection around kidney and identifying/ dissecting of the renal vessels and ureter were similar to pure LDN. The kidney was extracted manually via the Gelport.

Among donor factors, donor BMI was one of factors for the decision of procedures [11]. Hand-assisted LDN were more likely to be performed in patients with BMI ≥ 30. In contrast, LDN tended to be selected in patients with BMI < 30. Accordingly, the selection for LDN or hand-assisted LDN was decided by considering not only surgeon experience but also donor factors.

### Statistical analysis

To construct a difficulty grading system, univariate and multivariable analyses were performed to identify donor factors that were significantly associated with prolonged operative time. Factors associated with prolonged operative time were investigated with the Cox proportional hazards model showing odds ratio (OR) and 95% confidence interval (CI). The multivariable models to estimate surgical difficulty were used to generate a simple difficulty grading system [12], in which a score was assigned to each predictor based on the OR and a total score was corresponding to difficulty estimate. Finally, difficulty classification was categorized into three groups according to the difficulty index (total score): low, intermediate, and high difficulty. Data were presented as mean and standard deviation for continuous variables. Categorical data were presented as proportions. A P value < 0.05 was considered statistically significant. JMP version 11 software (SAS Institute, Cary, NC) was used for the statistical analyses.

## Results

The characteristics of the 1741 donors undergoing LDN between 1994 and 2018 are depicted in Table 1. Of these, 1320 (75.8%) were pure LDN and 421 (24.2%) were hand-assisted. The mean operative time and blood loss were 192 min and 171 mL, respectively. Overall conversion rate was 3.3% (*n* = 57). The incidence of postoperative major complications and mortality were 1.2% (*n* = 22) and 0%, respectively. Mean LOS was 3.4 days.

**Table 1:** Characteristics of laparoscopic donor nephrectomy between 1994 and 2018

Variables	Numbers
No. of patients	1741
Age (years)	51.5 (13.1)
Gender	
Male	760 (43.7%)
Female	995 (55.8%)
BMI (kg/m^2^)	26.3 (3.9)
Relationship	
Related	840 (48.2%)
Unrelated	556 (31.9%)
Cross over	224 (12.9%)
Non-directed	121 (7.0%)
Technique of LDN	
Pure	1320 (75.8%)
Hand-assisted	421 (24.2%)
Side of LDN	
Right	682 (38.2%)
Left	1101 (61.8%)
Operative time (minutes)	192 (72.6)
Blood loss (mL)	171 (274)
Conversion	57 (3.3%)
No. of renal artery (n = 1706)	
Single	1332 (80.0%)
Multiple	332 (20.0%)
No. of renal vein (n = 1706)	
Single	1511 (90.8%)
Multiple	153 (9.2%)
Postoperative major complications	22 (1.2%)
Length of stay (days)	3.4 (1.5)

*BMI* body mass index; *LDN* laparoscopic donor nephrectomy

### Donor factors associated with difficult laparoscopic donor nephrectomy

Univariate and multivariable analyses were carried out to explore which donor factors were associated with prolonged operative time (> 190 min). Multivariable analyses identified that male (OR 1.69, 95% CI 1.37–2.09, *P* < 0.001), BMI > 28 (OR 1.36, 95% CI 1.08–1.72, *P* = 0.009), pure LDN (OR 1.99, 95% CI 1.53–2.60, *P* < 0.001), multiple renal arteries (OR 2.38, 95% CI 1.83–3.10, *P* < 0.001) and multiple renal veins (OR 2.18, 95% CI 1.52–3.16, *P* < 0.001) were independent risk factors related to prolonged operative time (Table 2).

**Table 2:** Univariate and multivariable analysis associated with prolonged operative time (> 190 min)

Variables	Univariate			Multivariable		
	OR	95% CI	*P* value	OR	95% CI	*P* value
Age (years)						
≤ 60 (vs. > 60)	1.36	1.10–1.69	0.0052	1.20	0.96–1.52	0.12
Gender						
Male (vs. Female)	1.76	1.45–2.15	< 0.001	1.69	1.37–2.09	< 0.001
BMI (kg/m^2^)						
> 28 (vs. ≤ 28)	1.11	0.90–1.37	0.34	1.36	1.08–1.72	0.009
Technique of LDN						
Pure (vs. Hand-assisted)	2.11	1.67–2.69	< 0.001	1.99	1.53–2.60	< 0.001
Side of LDN						
Right (vs. Left)	1.39	1.14–1.70	0.001	1.09	0.87–1.36	0.4571
No. of renal artery						
Multiple (vs. Single)	2.59	2.02–3.34	< 0.001	2.38	1.83–3.10	< 0.001
No. of renal vein						
Multiple (vs. Single)	2.68	1.90–3.83	< 0.001	2.18	1.52–3.16	< 0.001

*BMI* body mass index; *LDN* laparoscopic donor nephrectomy; *OR* odds ratio; *CI* confidence interval

### A difficulty grading system of laparoscopic donor nephrectomy

**Fig. 1:**
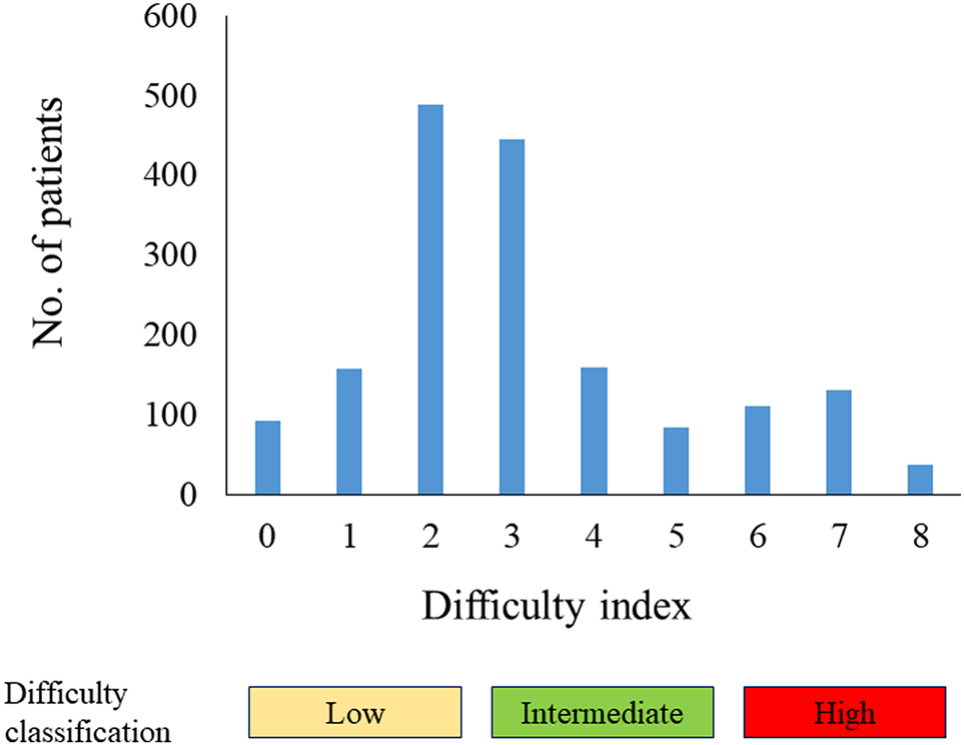
Distribution of the difficulty index

A difficulty grading system was applied for all patients, with 1 or 2 points to each significant donor factors based on a similar OR as in the multivariable analyses: 1 point to male, 1 point to BMI > 28, 2 points to pure LDN, 2 points to multiple renal arteries, and 2 points to multiple renal veins (Table 3). A total score of assigned points was defined as a difficulty index of LDN in the present study. The distribution of the difficulty index was represented in Fig. 1. The most frequent total score was with 2 points (28.5%) and 3 points (25.8%). Using the difficulty index of 0–8, indexes 0 and 8 were defined as the easiest and the most difficult case. Difficulty index was further categorized into three levels as the difficulty classification: low difficulty (0–2), intermediate difficulty (3–5), and high difficulty (6–8).

**Table 3:** A difficulty grading system of living donor nephrectomy

Variables	OR (95% CI)	Score
Gender		
Female	1 (reference)	0
Male	1.69 (1.37–2.09)	1
BMI (kg/m^2^)		
≤ 28	1 (reference)	0
> 28	1.36 (1.08–1.72)	1
Technique of LDN		
Hand-assisted	1 (reference)	0
Pure	1.99 (1.53–2.60)	2
No. of renal artery		
Single	1 (reference)	0
Multiple	2.38 (1.83–3.10)	2
No. of renal vein		
Single	1 (reference)	0
Multiple	2.18 (1.52–3.16)	2

Difficulty index (Total score)	0–8
Difficulty classification based on difficulty index	
Low	0–2
Intermediate	3–5
High	6–8

*BMI* body mass index; *LDN* laparoscopic donor nephrectomy; *OR* odds ratio; *CI* confidence interval

Outcomes based on the difficulty classification were shown in Table 4. Operative time was significantly longer in the high difficulty group (225 min) than in the low difficulty group (169 min, *P* < 0.001) and the intermediate difficulty group (194 min, *P* < 0.001). The incidence of conversion rate was higher in the high difficulty group (4.4%) than in the low (2.1%, *P* = 0.04) and the intermediate difficulty group (3.0%, *P* = 0.27). No significant difference in major complications was found between the groups.

**Table 4:** Outcomes based on the difficulty classification

Variables	Difficulty classification			P value
	Low (n = 725)	Intermediate (n = 668)	High (n = 271)	
Operative time (minutes)	169 (59.9)	194 (69.4)	225 (75.9)	< 0.001^a^
				< 0.001^b^
Blood loss (mL)	158 (213)	148 (250)	213 (391)	0.22^a^
				0.06^b^
Conversion	15 (2.1%)	20 (3.0%)	12 (4.4%)	0.04^a^
				0.27^b^
Major complications	8 (1.1%)	10 (1.5%)	4 (1.5%)	0.63^a^
				0.98^b^
Length of stay (days)	3.5 (1.5)	3.3 (1.3)	3.5 (1.6)	0.57^a^
				0.009 ^b^

^a^low vs high, and ^b^intermediate vs high

### Benchmark operation of laparoscopic donor nephrectomy

**Fig. 2:**
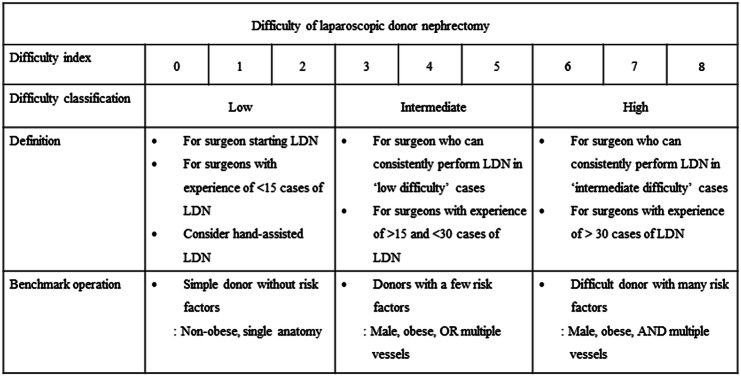
Benchmark operation of laparoscopic donor nephrectomy based on the difficulty index. *LDN* laparoscopic donor nephrectomy

Based on the difficulty index and the difficulty classification, we propose the benchmark operation of LDN, as demonstrated in Fig. 2. Simple donors classified with low difficulty are suitable for surgeons who start LDN and have low experiences of LDN. Hand-assisted LDN should be considered at this stage [13, 14]. Surgeons who consistently perform LDN in low difficulty cases or have 10–30 experiences of LDN can perform LDN in donors with a few risk factors classified with intermediate difficulty. LDN in difficult donors with many risk factors should be performed by surgeons with enough experiences of LDN.

## Discussion

The present study demonstrates a novel difficulty grading system for LDN by analyzing 1741 living donors in a high-volume center. Our difficulty grading system was developed by assessing easily available preoperative donor factors such as gender, BMI, the technique of LDN, and the number of renal artery and vein. The difficulty classification according to the difficulty index can predict the estimated operative time and the risk of conversion. In contrast, the incidence of postoperative major complications was similar regardless of the difficulty classification. In addition, we proposed the benchmark operations based on the difficulty classification evaluated by the difficulty index.

Several difficulty indexes, consisting of patient characteristics, anatomy, and tumor characteristics, have been used to classify the difficulty of several laparoscopic procedures [5–8]. Operative time has been reported to be a useful factor reflecting technical difficulty of laparoscopic procedures [15, 16], therefore we established a grading system to predict the difficulty of LDN based on multivariable analyses investigating preoperative donor factors associated with prolonged operative time. A recent study investigating operative difficulty of LDN using preoperative imaging has reported that perirenal fat around the donor kidney was significantly associated with operative time [9]. However, the measurement of perirenal fat using axial computed tomography imaging may show different results depending on the measurement level and inter-observer variability. In their study, perirenal fat has been reported to have a correlation with BMI, therefore the measurement of perirenal fat using preoperative imaging might be replaced by BMI. Actually, donor BMI was found to be one of factors that were significantly associated with operative time in the present study.

The benchmark operations of LDN were constructed based on previous reports and our experiences. The number of procedures requiring to perform LDN proficiently and independently remains controversial, therefore we defined inexperienced surgeons as performing < 15 cases of LDN, and experienced surgeons as performing > 30 cases according to the previous reports [17, 18]. Definition of each difficulty classification was constructed based on our experiences. Although specific technical skills and knowledge are essential in performing DN safely, we believe that our proposed benchmark operation of LDN can help in patient selection and in guiding surgeons especially with initial experiences of LDN. Our results based on the difficulty classification suggested that LDN in difficult donors with many risk factors should be performed by experienced surgeons for the donor safety. In contract, simple donors could be good candidates for inexperienced surgeons who start LDN and have low experiences of LDN.

A critical question is how to design training in the most efficient manner possible, while developing excellent skills and long-term retention. The graduated responsibility in surgical training is built on a balance between mastery of skills and the ability to recognize potential pitfalls. A recent review provides an evidence-based overview to optimize the learning curve [19]. An optimized dosage of delivering the training is important as well as setting a proficiency-based benchmark of performance. Retention of training effects and transfer from trained to non-trained domains depend on factors such as deliberate practice, part-task training, task variability, mental imagery, and overlearning after reaching proficiency. Distributing practice over time (spacing) leads to superior learning for knowledge acquisition, as well as motor skill acquisition compared to massed training. Our novel grading system in a high-volume center facilitates many factors to optimize the learning curve, such as proficiency-based benchmark of performance, part-task training, and distributing practice over time.

The present study has several limitations to be disclosed. We performed a retrospective study in a prospective database in a single center. There might be a potential donor selection bias. Regarding the selection for the technique of LDN, it was decided by considering not only surgeon experience but also donor factors. However, the selection was not randomly assigned, therefore an invariability might be created in the analysis. Although conversion has been used as a predictor of difficulty score among several operative surrogates in other fields [20, 21], this study selected operative time as an index of difficulty in LDN due to a low incidence of conversion in our study. The best parameter reflecting surgical difficulty is still controversial. Finally, our novel difficulty grading system was developed using a large single-center cohort, however the validation of the grading system was not carried out in the present study. Therefore the grading system should be validated to prove its significance in prospective multicenter cohorts. Further large studies are necessary to confirm the efficiency of this grading system on outcomes in LDN.

## Conclusions

We proposed a novel grading system with simple preoperative donor factors to predict the difficulty of LDN. Our difficulty index well reflects the estimated operative time and can easily categorize LDN as low, intermediate, or high difficulty. Moreover, this grading system can be helpful to guide surgeons in patient selection as well as advancing their experiences and/or teach fellows from simple to difficult LDN in a clinical practice. However, this system should be prospectively validated.
